# Supplementary material for: Targeted Next-Generation Sequencing Analysis Predicts the Recurrence in Resected Lung Adenocarcinoma Harboring EGFR Mutations
Source: Cancers (Basel). 2021 Jul 20;13(14):3632. doi: 10.3390/cancers13143632 (PMC8306820; doi:10.3390/cancers13143632)
Supplement: Supplementary file 1 [file cancers-13-03632-s001.zip › cancers-1262449-supplementary.pdf]

Table S1. Frequency and recurrence rate according to EGFR mutation subtypes.

EGFR mutation subtype	Case Number	Recurrence	Percentage (%)
**19 deletion**	56	17	30.3
p.E746_A750del	39	13	33.3
p.L747_P753delinsS	6	4	66.6
p.E746fs	2	0	0
p.E746fs+p.E747fs	2	0	0
p.E746_T751delinsA	4	0	0
p.L747_E749del	1	0	0
p.S752_I759del	1	0	0
p.L747fs+p.L751fs	1	0	0
**21 L858R mutation**	60	11	18.3
p.L858R	58	10	17.2
p.L861Q	2	1	50
**Exon 20 mutation**	5	2	40
p.S768_D770dup	3	1	33.3
p.N771_P772insH	1	0	0
p.A767_V769dup	1	1	100
**Double EGFR mutation**	10	3	30
19p.L747_E749del+19p.A750P	2	2	100
19p.D761N+19p.L747_P753delinsS	1	0	0
21p.L858R+19p.E746fs	1	0	0
20p.T790M+21p.L858R	1	1	100
21p.L858R+21p.V843I	1	0	0
18p.E709K+21p.L858R	1	0	0
21p.L861Q+18p.G719S	1	0	0
21p.L858R+20p.V819V	1	0	0
18p.G719S+20p.S768I	1	0	0
18p.E709K+18p.G719C	1	0	0

**Total**	**131**	**32**	**24.4**

A

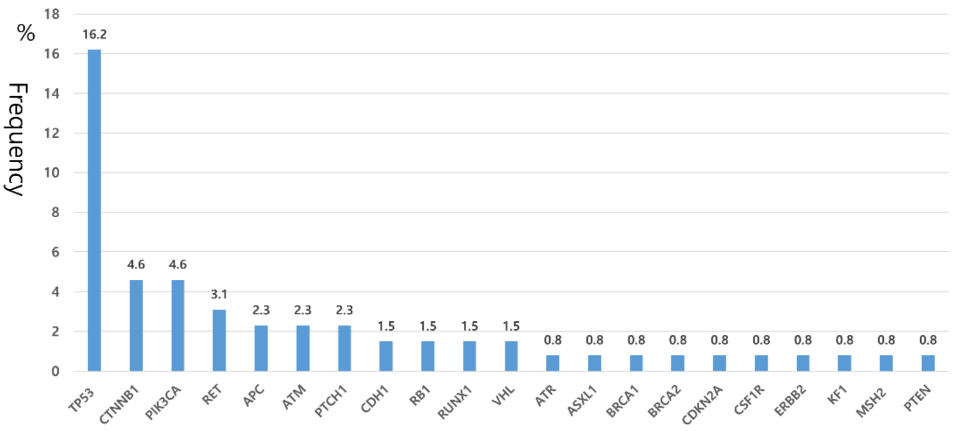

Figure S1. Frequency of co-occurring mutations in *EGFR*-mutated lung cancer specimens

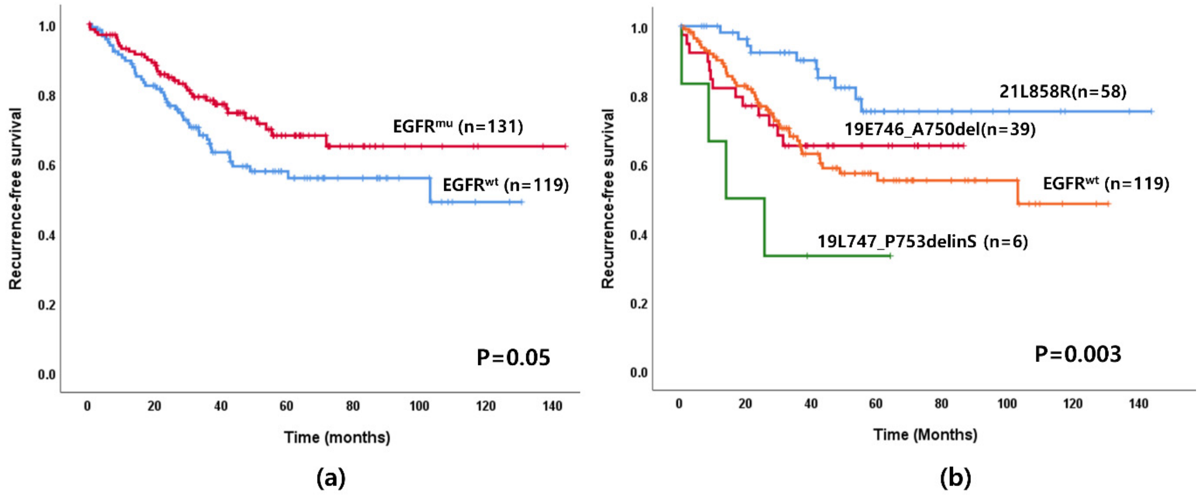

Figure S2. (a) Comparison of RFS between *EGFR* mutation and non-mutated *EGFR* cases (*EGFR*^{wt}) in resected lung adenocarcinoma. (b) Comparison of RFS among *EGFR*^{wt}, *EGFR*^{L858R}, *EGFR*$^{E746\_A750del}$ and *EGFR*$^{L747\_P753delinS}$ mutation.
